# The Caveolin-1 Scaffolding Domain Peptide Decreases Phosphatidylglycerol Levels and Inhibits Calcium-Induced Differentiation in Mouse Keratinocytes

**DOI:** 10.1371/journal.pone.0080946

**Published:** 2013-11-13

**Authors:** Haixia Qin, Wendy B. Bollag

**Affiliations:** 1 Charlie Norwood VA Medical Center, Augusta, Georgia, United States of America; 2 Department of Physiology, Medical College of Georgia at Georgia Regents University, Augusta, Georgia, United States of America; 3 Department of Medicine (Dermatology), Medical College of Georgia at Georgia Regents University, Augusta, Georgia, United States of America; 4 Departments of Orthopaedic Surgery, Oral Biology and Cell Biology and Anatomy, Georgia Regents University, Augusta, Georgia, United States of America; Cornell University, United States of America

## Abstract

Phospholipase D2 (PLD2) has been found localized in low-density caveolin-rich membrane microdomains. Our previous study suggested that PLD2 and aquaporin 3 (AQP3) interact in these domains to inhibit keratinocyte proliferation and promote differentiation by cooperating to produce phosphatidylglycerol. To examine the effect of membrane microdomain localization on the PLD2/AQP3 signaling module and keratinocyte proliferation and differentiation, we treated mouse keratinocytes with 3 µM cell-permeable caveolin-1 scaffolding domain peptide or a negative control peptide and stimulated cell differentiation using a moderately elevated extracellular calcium concentration (125 uM) to maximally promote differentiation and phosphatidylglycerol production. Cell proliferation, differentiation, total PLD activity, phosphatidylglycerol levels, and AQP3 activity were monitored. The caveolin-1 scaffolding domain peptide itself had no effect on phosphatidylglycerol levels or keratinocyte proliferation or differentiation but prevented the changes induced by a moderately elevated calcium concentration, whereas a negative control did not. The caveolin-1 scaffolding domain peptide had little effect on total PLD activity or glycerol uptake (AQP3 activity). We conclude that the caveolin-1 scaffolding domain peptide disrupts the functional association between AQP3 and PLD2 and prevents both the inhibited proliferation and the stimulated differentiation in response to elevated extracellular calcium levels. The interaction of caveolin-1 and PLD2 is indirect (i.e., lipid mediated); together with the proliferation-promoting effects of caveolin-1 knockout on epidermal keratinocytes, we propose that the caveolin-1 scaffolding domain pepetide exerts a dominant-negative effect on caveolin-1 to alter lipid rafts in these cells.

## Introduction

The epidermis is composed of several cell layers, with the basal layer continuously proliferating to replace cells lost to the environment in the uppermost layer. When the cells migrate up through the epidermis, they undergo a distinct pattern of differentiation. With this programmed proliferation and differentiation, the epidermis performs its function as the barrier of the skin [1]. Although much research has been performed to elucidate the regulation of this proliferation and differentiation process, the exact regulators and signaling mechanism(s) underlying these events are still largely unknown. However, one agent thought to be a key modulator of keratinocyte differentiation is the extracellular calcium concentration. Thus, *in vitro* keratinocytes maintain a basal phenotype and proliferate in low extracellular calcium levels and differentiate upon exposure to elevated calcium concentrations (reviewed in 2). Corresponding to this *in vitro* situation, in the epidermis *in situ* a calcium gradient has been observed, with low levels detected in the basal layer and higher levels measured in the suprabasal layers, using ion-capture cytochemistry [3-5]. In keratinocytes extracellular calcium binds to and activates the calcium-sensing receptor in a concentration-dependent manner [6]. Through a heterotrimeric G protein, the calcium-bound receptor then stimulates phospolipase C-β and -γ, which hydrolyze phosphatidylinositol 4,5-bisphosphate to diacylglycerol and inositol 1,4,5-trisphosphate (IP_3_) [7,8]. IP_3_ combines with the IP_3_ receptor on intracellular calcium stores, leading to the release of this calcium and an increase in the intracellular calcium level, which then triggers keratinocyte differentiation ([9] and reviewed in 2).

The aquaporins (AQPs) are a family of small (~30 kDa/monomer), hydrophobic, integral membrane proteins that transport water and in some cases small solutes. So far, 13 members have been identified in mammals [10,11]. According to their structural and functional properties, aquaporins can be divided into two subgroups: ‘‘aquaporins’’, which transport only water, and ‘‘aquaglyceroporins’’, which can transport both water and glycerol [12-14]. AQP3 is categorized as an aquaglyceroporin and transports glycerol as well as water [15]. AQP3 is expressed in the basal layer of keratinocytes in mammalian skin [11,16,17], as well as in the suprabasal layers ([18] and reviewed in 19). Mice lacking AQP3 have dry skin with reduced stratum corneum hydration, decreased elasticity and impaired lipid biosynthesis and barrier recovery [16]. Reduced transport of glycerol from the blood to the keratinized layer is observed in these AQP3-null mice [20], and it is the selectively reduced glycerol in the skin of AQP3-deficient mice, rather than decreases in water transport, that likely account for the epidermal abnormalities [21]. Indeed, glycerol administration can correct the epidermal phenotypes [20], suggesting the importance of glycerol in keratinocyte physiology. 

PLD is thought to be involved in a variety of cellular responses, including cell proliferation and differentiation [22,23]. Mammalian PLD has two isoforms, namely PLD1 and PLD2, which have approximately 50% overall sequence homology [24]. In general, both PLDs can hydrolyze phosphatidylcholine to produce choline and phosphatidic acid, or in the presence of a primary alcohol, they catalyze a transphosphatidylation reaction to produce phosphatidylalcohols. Research in our laboratory has shown that PLD2, which localizes primarily to the plasma membrane [25], can utilize glycerol *in vitro* in the transphosphatidylation reaction to generate phosphatidylglycerol, which can be used as a method to measure PLD2 activity in intact keratinocytes [26]. In keratinocytes a modestly elevated extracellular calcium concentration (125 uM), which inhibits keratinocyte proliferation and promotes differentiation, increases phosphatidylglycerol levels in a PLD-mediated manner [26]. The colocalization of glycerol-transporting AQP3 with PLD2 in caveolin-rich membrane microdomains [27] provides a mechanism by which glycerol for phosphatidylglycerol production is supplied to PLD2. Furthermore, our data suggest that the phosphatidylglycerol generated by PLD2 and AQP3 can trigger early keratinocyte differentiation. Thus, glycerol, but not equivalent concentrations of sorbitol or xylitol, inhibits keratinocyte proliferation [28]. Similarly, phosphatidylglycerol liposomes inhibit keratinocyte proliferation, while those composed of phosphatidylpropanol do not [28]. Finally, coexpression of AQP3 with reporter constructs driven by promoters for keratinocyte markers indicate that AQP3 decreases the promoter activity of keratin 5, a basal (proliferative) marker, increases promoter activity of the early differentiation marker, keratin 1, and enhances the effect of increased calcium on the promoter activity of involucrin [28], a marker of intermediate differentiation. Together these results suggest a role for the PLD2/AQP3 signaling module in inhibiting proliferation and stimulating differentiation in epidermal keratinocytes.

 Caveolae are small (~50-100 nm) flask-shaped invaginations of the plasma membrane enriched in sphingolipids and cholesterol [29]. These dynamic structures may act as physically and biochemically distinct plasma membrane compartments that localize and regulate transmembrane signaling events [30-32]. Caveolae are formed from lipid rafts/membrane microdomains by polymerization of caveolins, which are hairpin-like palmitoylated integral membrane proteins that tightly bind cholesterol [30,31,33-35]. Three caveolin family members have been cloned and designated caveolin-1, caveolin-2 and caveolin-3 [36]. Among them, caveolin-1 has been shown to form homo-oligomers comprising ~14-16 monomers, as well as heterooligomers with caveolin-2 [37-39]. A small domain located in the N-terminus of caveolin-1, named the caveolin-1 scaffolding domain, has been shown to serve as a structural scaffold responsible for interactions with many proteins, including enzymes involved in call signaling [36]. In general, caveolin-1 interaction inhibits the activity of its binding partners, either holding them in an inactive state or terminating activity following a stimulus. Molecules that have been found to be associated with caveolin-1/caveolae in various cell types include Ras, Src family tyrosine kinases, G proteins, G-protein-coupled receptors, endothelial nitric oxide synthase, the sonic hedgehog receptor patched, serine/threonine kinases, the epidermal growth factor receptor, cyclooxygenase-2, neutral sphingomyelinase, and protein kinase A [40-45]. In addition, PLD2 activity has been shown to be regulated by caveolin-1 [46,47]. Importantly, the caveolin scaffolding domain is also involved in oligomerization of caveolin.

Since AQP3 can transport glycerol, PLD2 can utilize glycerol to produce phosphatidylglycerol, and both of them colocalize in caveolin-rich membrane microdomains, we hypothesized that the caveolin-1 scaffolding domain peptide could inhibit the functional interaction of AQP3 and PLD2 and reduce phosphatidylglycerol production. Because phosphatidylglycerol acts as a lipid second messenger to inhibit keratinocyte growth and promote differentiation [28], we predicted that the caveolin-1 scaffolding domain peptide would also inhibit differentiation induced by a moderate elevation in extracellular calcium concentration (to 125 μM). 

## Materials and Methods

### Cell Culture

All experiments with animals were approved by the appropriate Institutional Animal Care and Use Committee (the Georgia Regents University IACUC and the Charlie Norwood VA Medical Center IACUC). The laboratory animal programs at Georgia Regents University and the Charlie Norwood VA Medical Center both are accredited by the Association for Assessment and Accreditation of Laboratory Animal Care International (AAALAC), are Registered Research Facilities with the United States Department of Agriculture (USDA) and have Office of Laboratory Animal Welfare (OLAW) Assurance Statements on file (A3307-01 and A3326-01, respectively). Epidermal keratinocytes were prepared from neonatal ICR CD1 mice according to the method of Yuspa and Harris [48] with the appropriate institutional approvals. Briefly, after trypsinization of the skin, the epidermis was mechanically separated from the dermis, and epidermal keratinocytes were released by scraping. Cells were plated at a density of 25,000 cells per cm^2^ and were allowed to attach overnight in keratinocyte plating medium, followed by refeeding with a low (25 µM) Ca^2+^ serum-free keratinocyte medium (SFKM), as described by Griner et al. [49]. Cells were stimulated to differentiate by a moderately elevated calcium concentration (125 µM), a concentration that induces essentially maximal protein expression of keratinocyte differentiation markers [50] and phosphatidylglycerol production [51].

### Measurement of DNA Synthesis

Near-confluent cultures were refed with SFKM containing 0.1% DMSO as the control (Con), 3 µM cell-permeable caveolin-1 scaffolding domain peptide (Calbiochem, Darmstadt, Germany), 3 µM cell-permeable caveolin-1 scaffolding domain peptide negative control (Neg, a scrambled version of the caveolin-1 scaffolding domain peptide), 125 µM CaCl_2_ [+ 0.1% DMSO (Ca)], 125 µM CaCl_2_ + 3 µM cell-permeable caveolin-1 scaffolding domain peptide (Ca + CSDP), and 125 µM CaCl_2_ + 3 µM cell-permeable caveolin-1 scaffolding domain peptide negative control (Ca + Neg). After 24 hours, cells were labeled with 1 µCi/mL [^3^H]thymidine for 1 h. Cultures were washed twice with phosphate-buffered saline without calcium or magnesium (PBS-) and macromolecules were precipitated using ice-cold 5% trichloroacetic acid (TCA). After additional washing with 5% TCA and distilled water, cells were solubilized in 0.3 M NaOH. An aliquot of this NaOH extract was counted in a liquid scintillation spectrometer.

### Measurement of Transglutaminase Activity

Near-confluent cultures were treated for 24 hours as described above. Cells were then scraped into homogenization buffer (0.1 M Tris-acetate, pH 7.8, 2 µg/ml aprotinin, 2 µM leupeptin, 1 µM pepstatin A, 0.2 mM EDTA and 0.2 µM 4-(2-aminoethyl)-benzenesulfonylfluoride hydrochloride), collected by centrifugation and subjected to one freeze-thaw cycle prior to disruption by sonication. Aliquots of the homogenate were removed for determination of protein content and transglutaminase activity. Transglutaminase activity was measured as [^3^H]putrescine incorporation into casein after an overnight incubation at 37°C. Casein was precipitated with TCA, collected onto glass fiber filters and counted by liquid scintillation spectrometry. The cellular protein content of the samples was determined using the Bio-Rad D_*C*_ protein assay system (Bio-Rad, Hercules, CA), with BSA as standard, and transglutaminase activity was calculated as cpm/µg protein and expressed relative to the control value.

### Measurement of Total Phospholipase D Activity in Intact Cells

Total PLD activity was assayed as detailed in [52]. Briefly, near-confluent cultures of primary keratinocytes were labeled for 24 h with [^3^H]oleic acid in 6 groups as described above. 0.5% Ethanol was then added for an additional 30 min. The incubations were terminated by aspirating the medium and lysing the cells by the addition of 0.2% SDS containing 5 mM EDTA. The radiolabeled phosphatidylethanol (PEt) was then extracted into chloroform/methanol, separated by thin-layer chromatography, visualized with autofluorography using En^3^Hance, and quantified by liquid scintillation spectrometry of the excised phospholipid bands, identified by comigration with authentic standards

### Measurement of Phosphatidylglycerol Levels in Intact Cells

Near-confluent keratinocytes were treated for 24 hours in 6 groups as described above, and 0.5 µCi/ml [^14^C]glycerol was then added for an additional 30 min. The incubations were terminated by aspirating the medium and lysing the cells by the addition of 0.2% SDS containing 5 mM EDTA. The radiolabeled phosphatidylglycerol was then extracted into chloroform/methanol, separated by thin-layer chromatography, visualized with autofluorography using En^3^Hance, excised, and quantified by liquid scintillation spectrometry as in [51]. For the calcium pre-differentiation experiments, the cells were pretreated with medium containing 125 µM calcium prior to exposure to vehicle (Con), the caveolin-1 scaffolding domain peptide, and the negative control peptide for 4h, 12 h or 20 h as indicated. Phosphatidylglycerol levels were measured as above.

### [^3^H]Glycerol Uptake

Radiolabeled glycerol uptake was monitored as in [51]. Briefly, near-confluent primary keratinocytes were treated for 24 h in 6 groups as described above. The medium was aspirated and replaced with SFKM containing 20 mM HEPES and 1 µCi per mL [^3^H]glycerol for exactly 5 minutes. Reactions were terminated by washing three times with ice-cold PBS-. The cells were subsequently solubilized in 0.3M NaOH and aliquots of this extract were subjected to liquid scintillation counting. 

### Inositol 1,4,5-Trisphosphate (IP_3_) Assay

Near-confluent keratinocytes pretreated with vehicle (0.1% DMSO) or 3 µM caveolin-1 scaffolding domain peptide for 24 hours, were incubated with 25 µM CaCl_2_ (Con) or 1 mM CaCl_2_ for exactly 10 minutes. This concentration of calcium was used to stimulate phosphoinositide hydrolysis to maximize the immediate response, as 1 mM calcium generates a greater change in intracellular calcium levels, and presumably triggering IP_3_ levels, than do lower calcium concentrations [6] and drives keratinocytes towards late differentiation [53]. Cells were then collected in 300µl PBS-. IP_3_ levels were measured with a radioreceptor assay kit according to the manufacturer’s directions (PerkinElmer, Boston MA). Any samples with a concentration of IP_3_ determined to be beyond the range of the standard curve were diluted with distilled water and re-assayed. The values obtained were then multiplied by the appropriate dilution factor.

### Statistical Analysis

The data are presented as means ± S.E.M. Each keratinocyte preparation represents a separate experiment. Data were analyzed using one-way analysis of variance (ANOVA) to compare various groups using the program Graphpad Prizm (La Jolla, CA), with significance accepted at p<0.05.

## Results

### The caveolin-1 scaffolding domain peptide prevents the inhibition of DNA synthesis and the stimulation of transglutaminase activity induced by a moderately elevated calcium level

**Figure 1:**
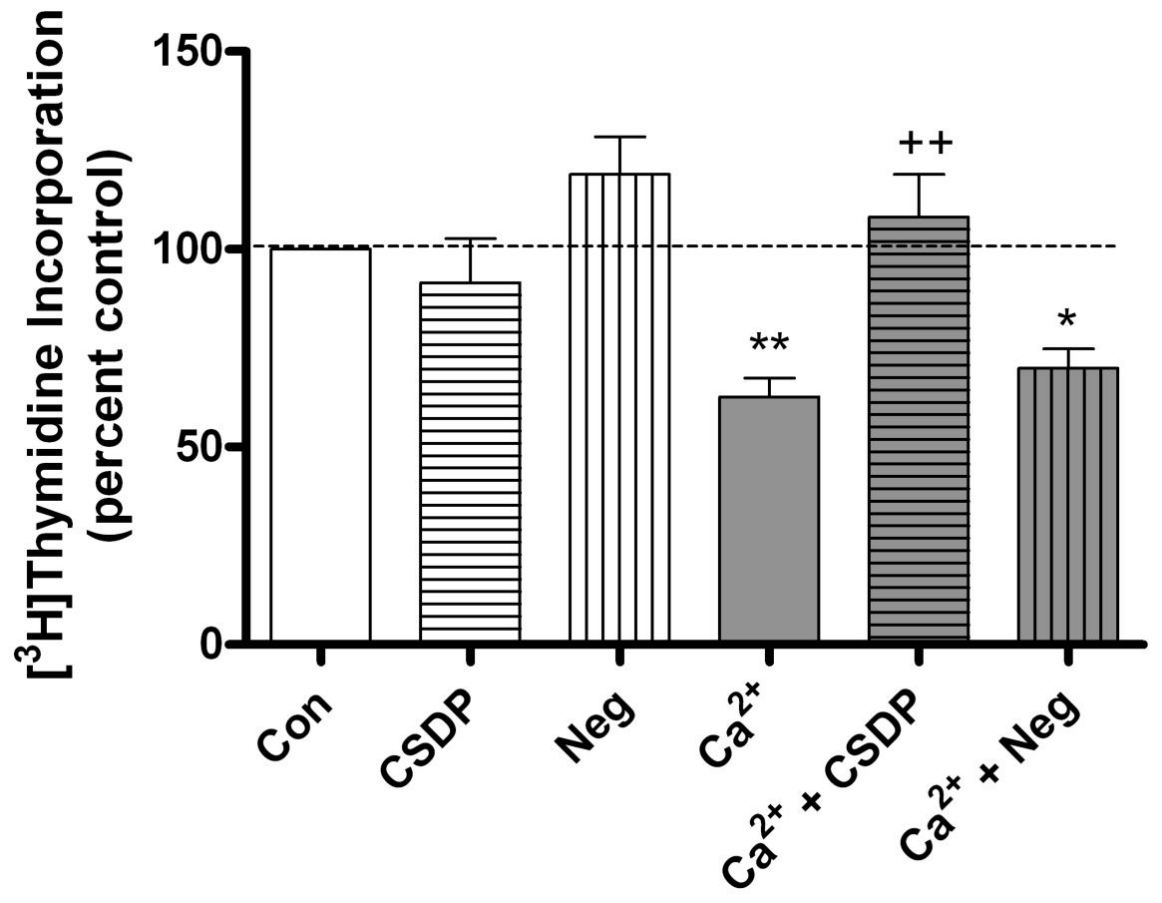
The caveolin-1 scaffolding domain peptide prevents the calcium-induced inhibition of DNA synthesis. Keratinocytes were treated for 24 hours with medium containing vehicle (0.1% DMS0) or 3 µM caveolin-1 scaffolding domain peptide (CSDP) or the negative control (Neg) in medium containing 25 µM calcium (Con) or 125 µM calcium (Ca^2+^), as indicated. [^3^H]Thymidine incorporation into DNA was measured as described in the Materials and Methods. Values are expressed as the percent control and represent the means ± SEM of 12 separate experiments performed in duplicate; *p<0.05, **p<0.01 versus the control value; ++p<0.01 versus calcium alone.

We hypothesized that the caveolin-1 scaffolding domain peptide would disrupt caveolin-1 actions and alter keratinocyte function. To study the influence of the caveolin-1 scaffolding domain peptide on the proliferation of near-confluent mouse keratinocytes, [^3^H]thymidine incorporation into DNA was performed in proliferating and differentiating keratinocytes. In this and the following experiments, a scrambled caveolin-1 scaffolding domain peptide was used as a negative control. This peptide has the same amino acid composition but in a different sequence compared to the caveolin-1 scaffolding domain peptide. Keratinocyte differentiation was stimulated by exposure of the cells to a moderately elevated calcium concentration, from 25 to 125 µM. As shown in Figure 1, after 24 hours of treatment, this moderately elevated calcium concentration inhibited DNA synthesis by approximately 40%, consistent with the ability of this agent to induce differentiation, one of the first steps of which is growth arrest. On the other hand, the caveolin-1 scaffolding domain peptide prevented this inhibition of DNA synthesis induced by 125 µM calcium (p<0.05), but the negative control had no such effect. 

**Figure 2:**
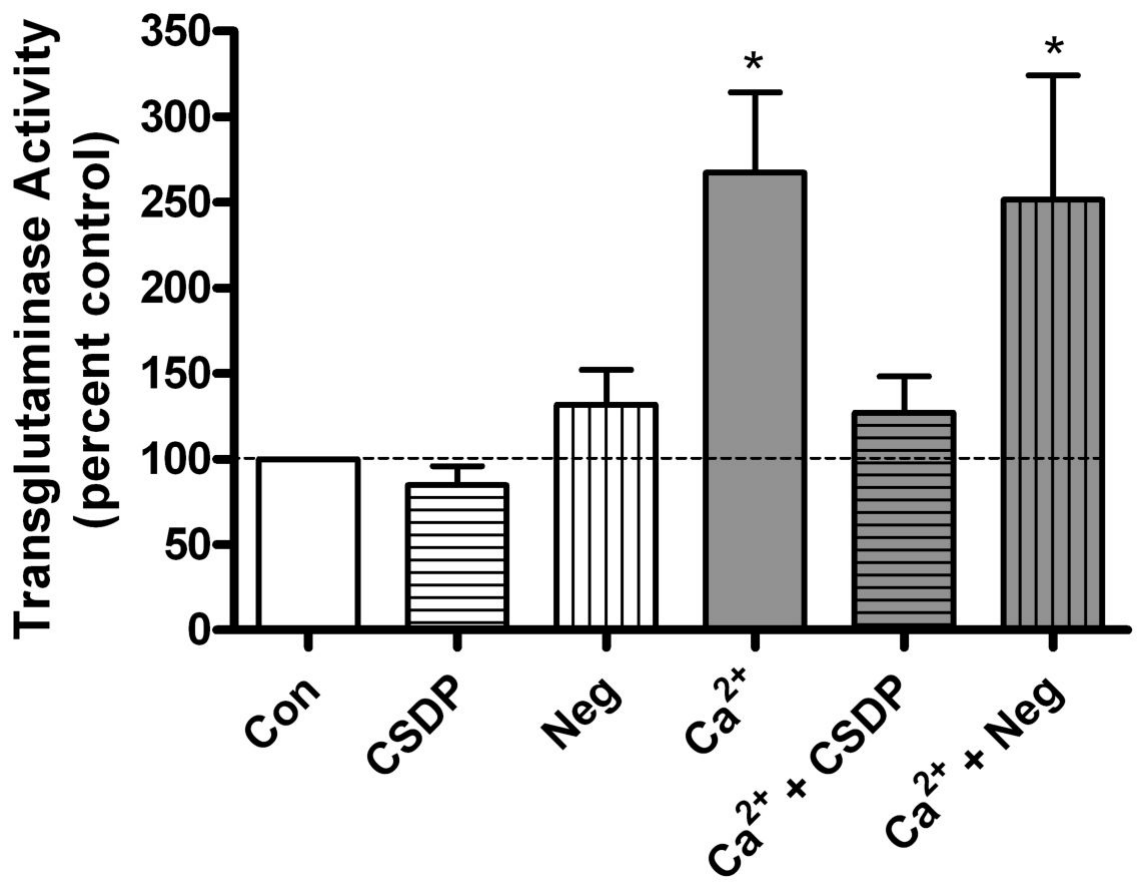
The caveolin-1 scaffolding domain peptide prevents the calcium-induced stimulation of transglutaminase activity. Keratinocytes were treated for 24 hours with SFKM containing vehicle (0.1% DMSO) or 3 µM caveolin-1 scaffolding domain peptide (CSDP) or the negative control (Neg) in medium containing 25 µM calcium (Con) or 125 µM calcium (Ca^2+^), as indicated. Transglutaminase activity was then measured as described in Materials and Methods. Values are expressed as the percent control and represent the means ± SEM of 6 separate experiments performed in duplicate; *p<0.05 versus the control value.

To determine the influence of the caveolin-1 scaffolding domain peptide on keratinocyte differentiation, transglutaminase, a late keratinocyte differentiation marker, was used to monitor keratinocyte differentiation. As seen in Figure 2, 125 µM calcium stimulated transglutaminase activity when compared with control, while the caveolin-1 scaffolding domain peptide returned transglutaminase activity to a level not significantly different from the control value. Again, the negative control had no effect on calcium’s induction of transglutaminase activity. These results indicated that the caveolin-1 scaffolding domain peptide inhibited differentiation in response to an elevated calcium level, suggesting a possible effect of this peptide on calcium-induced signaling processes. 

### The caveolin-1 scaffolding domain peptide does not inhibit the initial calcium signaling pathway

**Figure 3:**
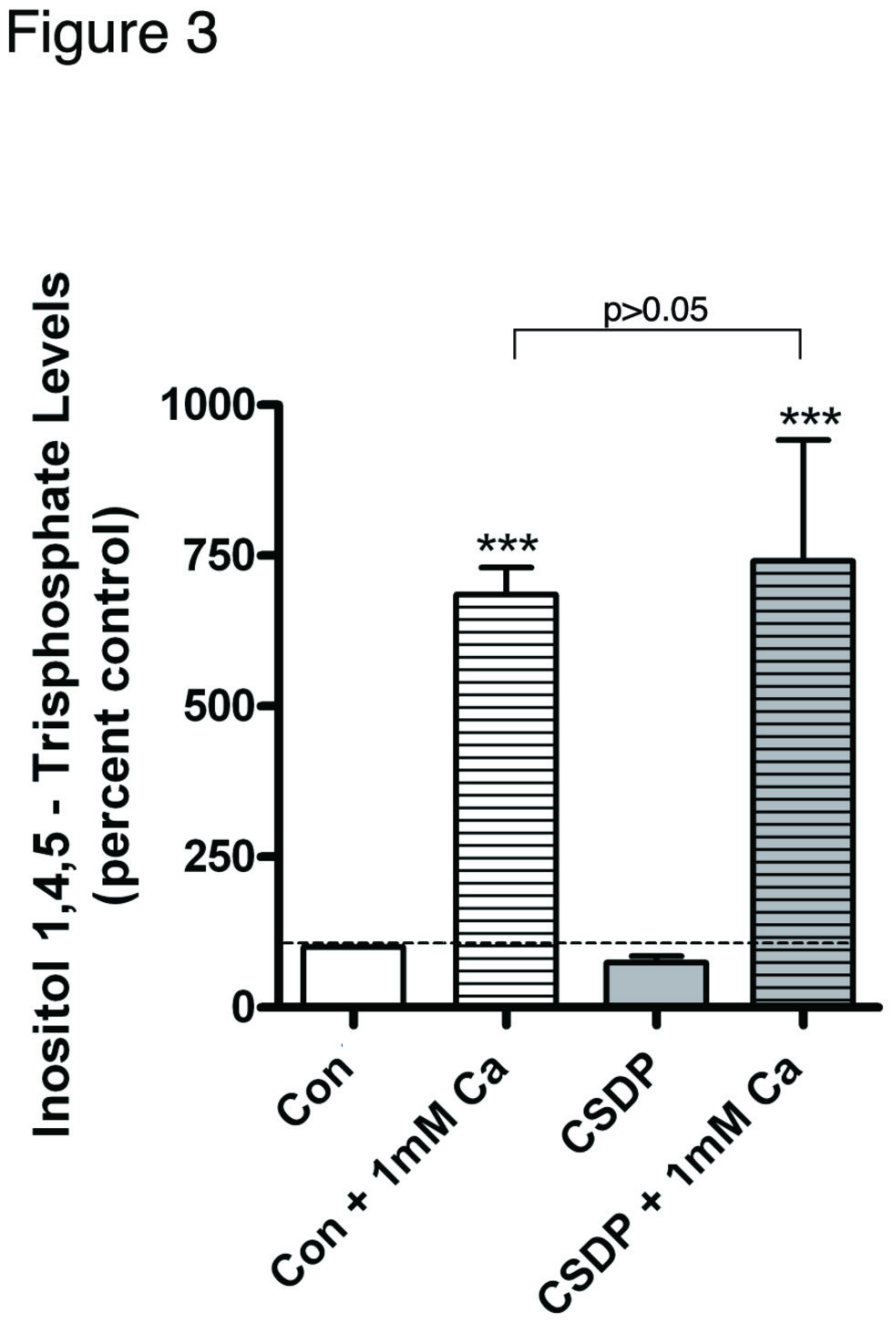
Caveolin-1 scaffolding domain peptide pretreatment has no effect on calcium-induced IP_3_ production. Keratinocytes were pretreated for 24 hours with SFKM containing vehicle (0.1% DMS0) or 3 µM caveolin-1 scaffolding domain peptide (CSDP). The cells were then treated for 10 minutes with control (25 µM calcium-containing) medium or 1 mM calcium-containing medium (to trigger immediate and maximal calcium-sensing receptor activation), and inositol 1,4,5-trisphosphate levels were measured with a radioreceptor assay as described in Materials and Methods. Values are expressed as the percent control and represent the means ± SEM of 4 separate experiments performed in duplicate; ***p<0.001 versus the control value.

As the caveolin-1 scaffolding domain peptide inhibited keratinocyte differentiation upon stimulation with 125 µM calcium, whether or not the caveolin-1 scaffolding domain peptide inhibited reception of the calcium signal, i.e. activation of the calcium-sensing receptor by calcium, had to be considered. Therefore, we pretreated keratinocytes with 3 µM caveolin-1 scaffolding domain peptide or vehicle for 24 hours, followed by treatments with control medium (25 µM calcium) or medium containing 1 mM calcium for 10 minutes and measurement of IP_3_ levels. This concentration of calcium was used to stimulate phosphoinositide hydrolysis to maximize the immediate response, as discussed in Methods. As shown in Figure 3, a 10-minute stimulation of keratinocytes with 1 mM calcium resulted in a significant increase in IP_3_ levels in both the vehicle and 3 µM caveolin-1 scaffolding domain peptide-pretreated cells, and more importantly, there was no significant difference in IP_3_ production between the two groups. This result indicates that the caveolin-1 scaffolding domain peptide does not inhibit initiation of the calcium signaling pathway. 

### The caveolin-1 scaffolding domain peptide completely inhibits calcium-induced phosphatidylglycerol production, but has minimal effects on radiolabeled phosphatidylethanol (PEt) levels and glycerol uptake

**Figure 4:**
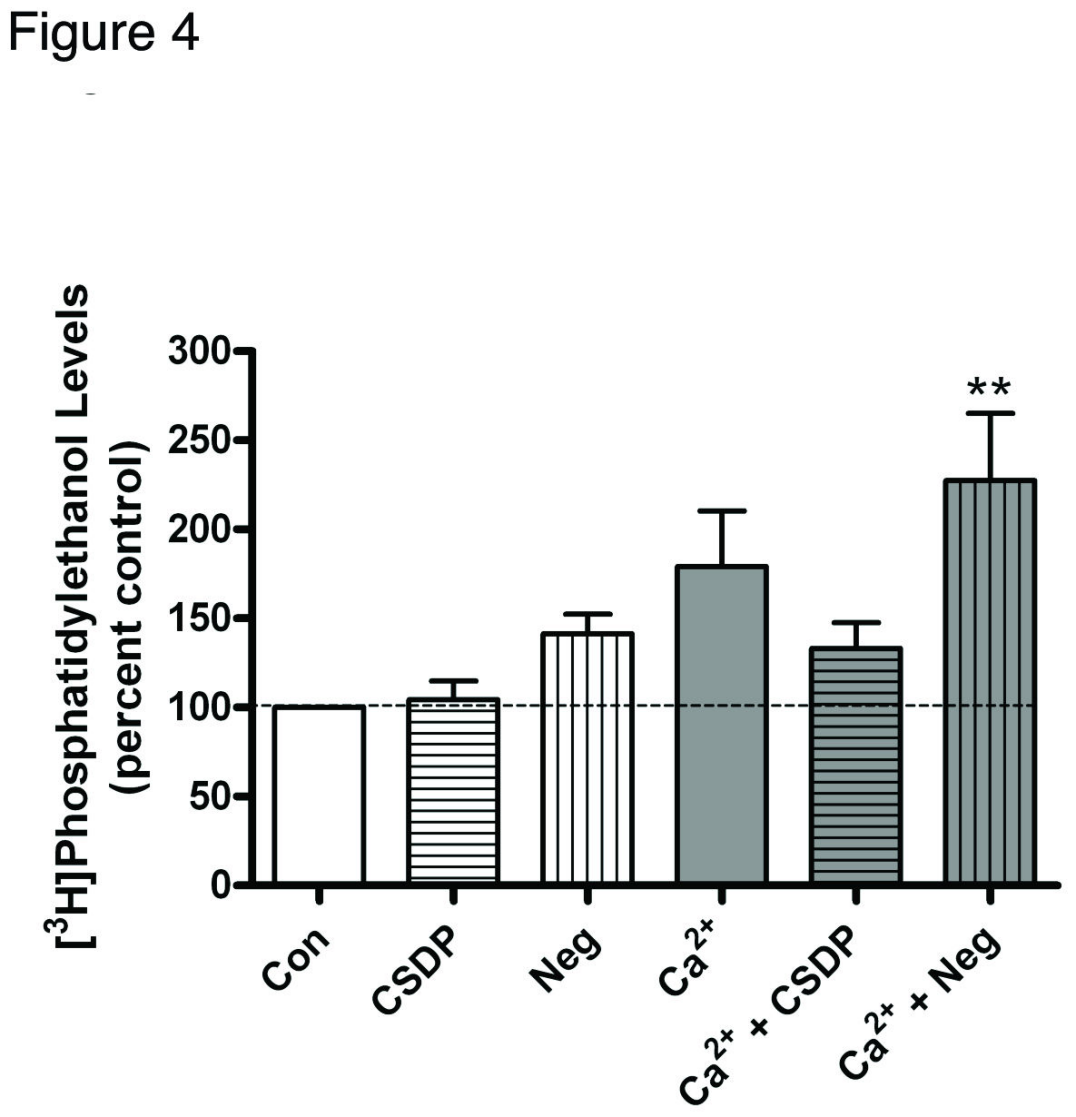
The caveolin-1 scaffolding domain peptide has a minimal effect on calcium-induced PLD activation. Keratinocytes were preincubated for 24 hours with SFKM containing [^3^H]oleate and vehicle (0.1% DMS0), 3 μM caveolin-1 scaffolding domain peptide (CSDP) or the negative control (Neg) in medium containing 25 µM calcium (Con) or 125 µM calcium (Ca^2+^) as indicated. [^3^H]Phosphatidylethanol levels were then measured as described in Materials and Methods. Values are expressed as the percent control and represent the means ± SEM of 5 separate experiments performed in duplicate; **p<0.01 versus the control value.

**Figure 5:**
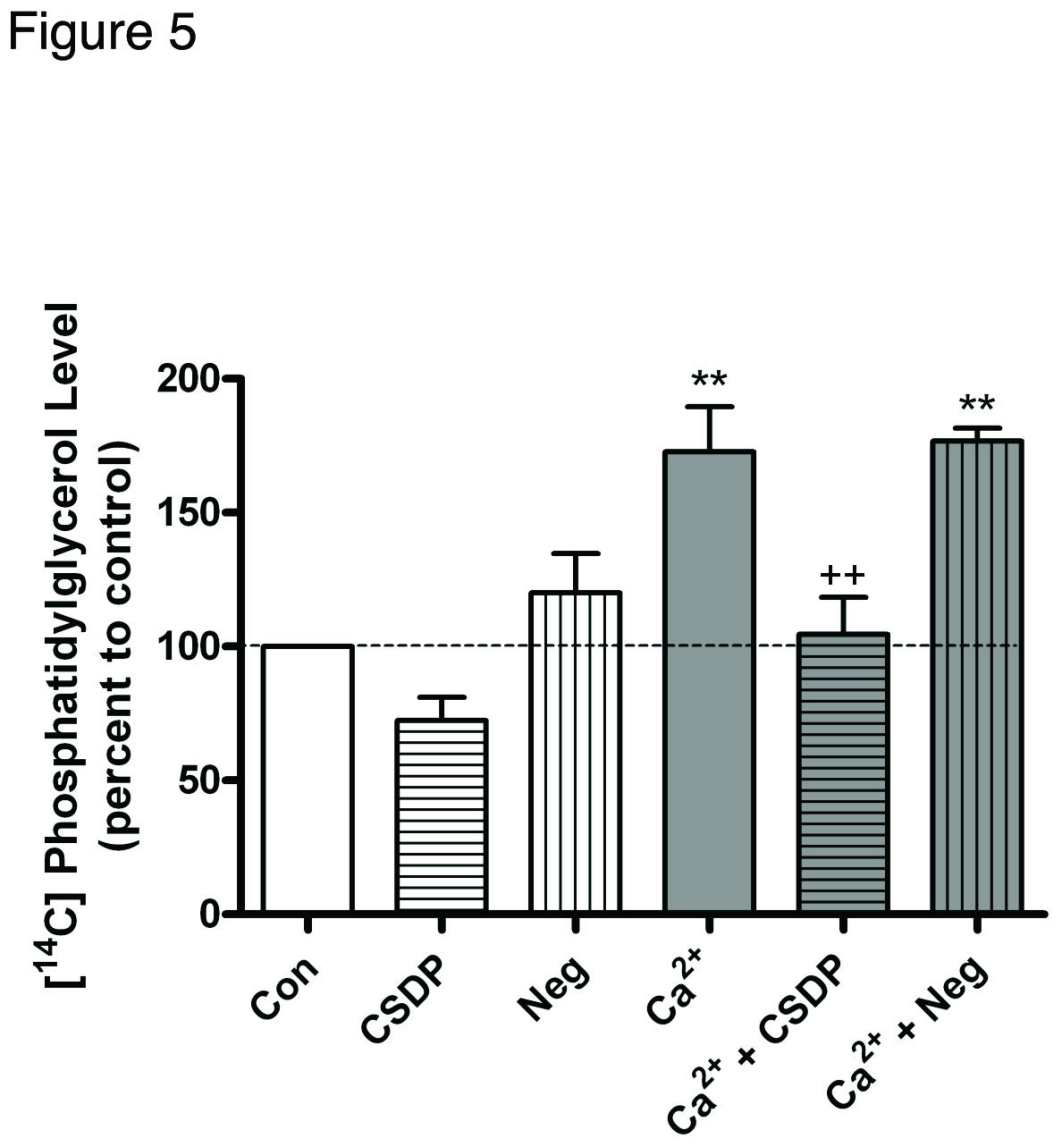
The caveolin-1 scaffolding domain peptide decreases calcium-increased phosphatidylglycerol levels. Keratinocytes were treated for 24 hours with SFKM containing vehicle (0.1% DMSO) or 3 μM caveolin-1 scaffolding domain peptide (CSDP) or the negative control (Neg) in medium containing 25 μM calcium (Con) or 125 μM calcium (Ca^2+^) as indicated.. [^14^C]Phosphatidylglycerol levels were then measured as described in Materials and Methods. Values are expressed as the percent control and represent the means ± SEM of 3 separate experiments performed in duplicate; **p<0.01 versus the control value; ††p<0.01 versus calcium alone.

The caveolin-1 scaffolding peptide did not affect the initiation of calcium signaling but prevented the calcium-induced inhibition of keratinocyte proliferation and promotion of differentiation. One pathway activated by a moderately elevated calcium concentration is the PLD2/AQP3 signaling module, and our data suggest the involvement of this module in promoting early keratinocyte differentiation. Therefore, we hypothesized that the caveolin-1 scaffolding peptide might affect the functional interaction of these two proteins, thereby preventing the production of phosphatidylglycerol and keratinocyte differentation in response to an elevated calcium concentration. We first examined the effect of the caveolin-1 scaffolding peptide on PLD activity. Total PLD activity and phosphatidylglycerol levels (a measure of PLD2 activity) were monitored by determining the effects of the caveolin-1 scaffolding domain peptide on [^3^H]phosphatidylethanol and [^14^C]phosphatidylglycerol levels, respectively, in control and moderately elevated calcium-containing medium. As shown in Figure 4, total PLD activity changed little in response to calcium and/or treatment with the caveolin-1 scaffolding domain peptide. Indeed, only the combination of calcium and the negative control peptide induced a statistically significant increase in radiolabeled phosphatidylethanol levels. On the other hand, phosphatidylglycerol levels increased in response to calcium stimulation, and this effect was completely prevented by the caveolin-1 scaffolding domain peptide (Figure 5). This result suggests the possible involvement of inhibition of phosphatidylglycerol production in the changes in keratinocyte proliferation and differentiation observed with the caveolin-1 scaffolding domain peptide. 

**Figure 6:**
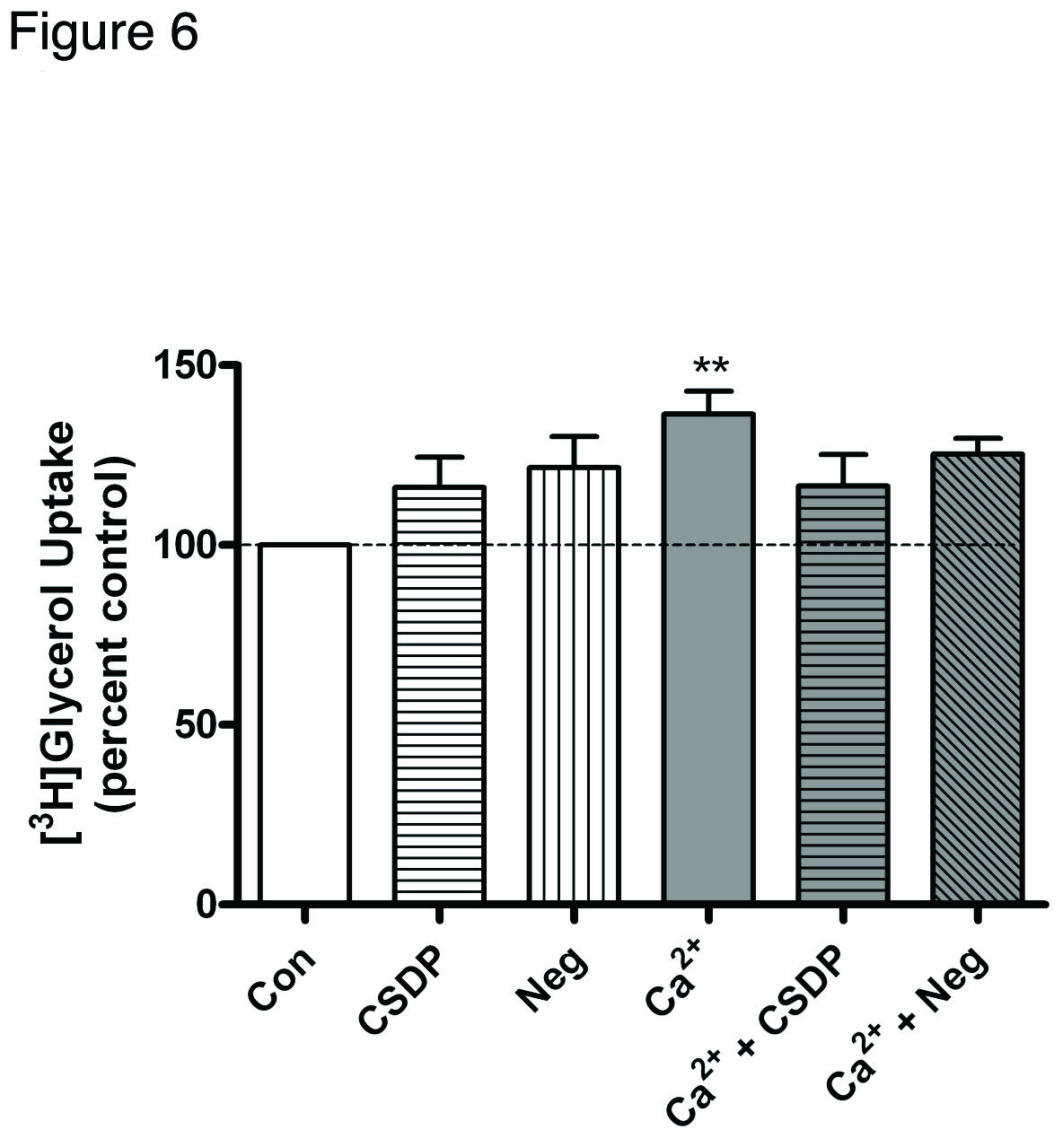
The caveolin-1 scaffolding domain peptide has a minimal effect on calcium-induced glycerol uptake. Keratinocytes were treated for 24 hours with SFKM containing vehicle (0.1% DMS0) or 3 µM caveolin-1 scaffolding domain peptide (CSDP) or the negative control (Neg) in medium containing 25 µM calcium (Con) or 125 µM calcium (Ca^2+^) as indicated. [^3^H]Glycerol uptake was then measured as described in Materials and Methods. Values are expressed as the percent control and represent the means ± SEM of 4 separate experiments performed in duplicate; **p<0.01 versus the control value.

As AQP3 has been shown to be important for transporting glycerol into mouse keratinocytes (and to PLD2), it was important to examine the change in AQP3 activity upon treatment with calcium and/or the caveolin-1 scaffolding domain peptide, as measured by [^3^H]glycerol uptake. As shown in Figure 6, calcium treatment increased glycerol uptake, while the caveolin-1 scaffolding domain peptide had a minimal effect on this calcium-induced uptake. Because the caveolin-1 scaffolding domain peptide had minimal effects on AQP3 activity and total PLD activity, we suspected that the proliferation and differentiation changes in mouse keratinocytes treated with the caveolin-1 scaffolding domain peptide could be the result, at least in part, of decreased phosphatidylglycerol levels, possibly resulting from a disruption in the functional interaction of PLD2 and AQP3. 

### Phosphatidylglycerol levels are decreased by the caveolin-1 scaffolding domain peptide after differentiation is triggered

**Figure 7:**
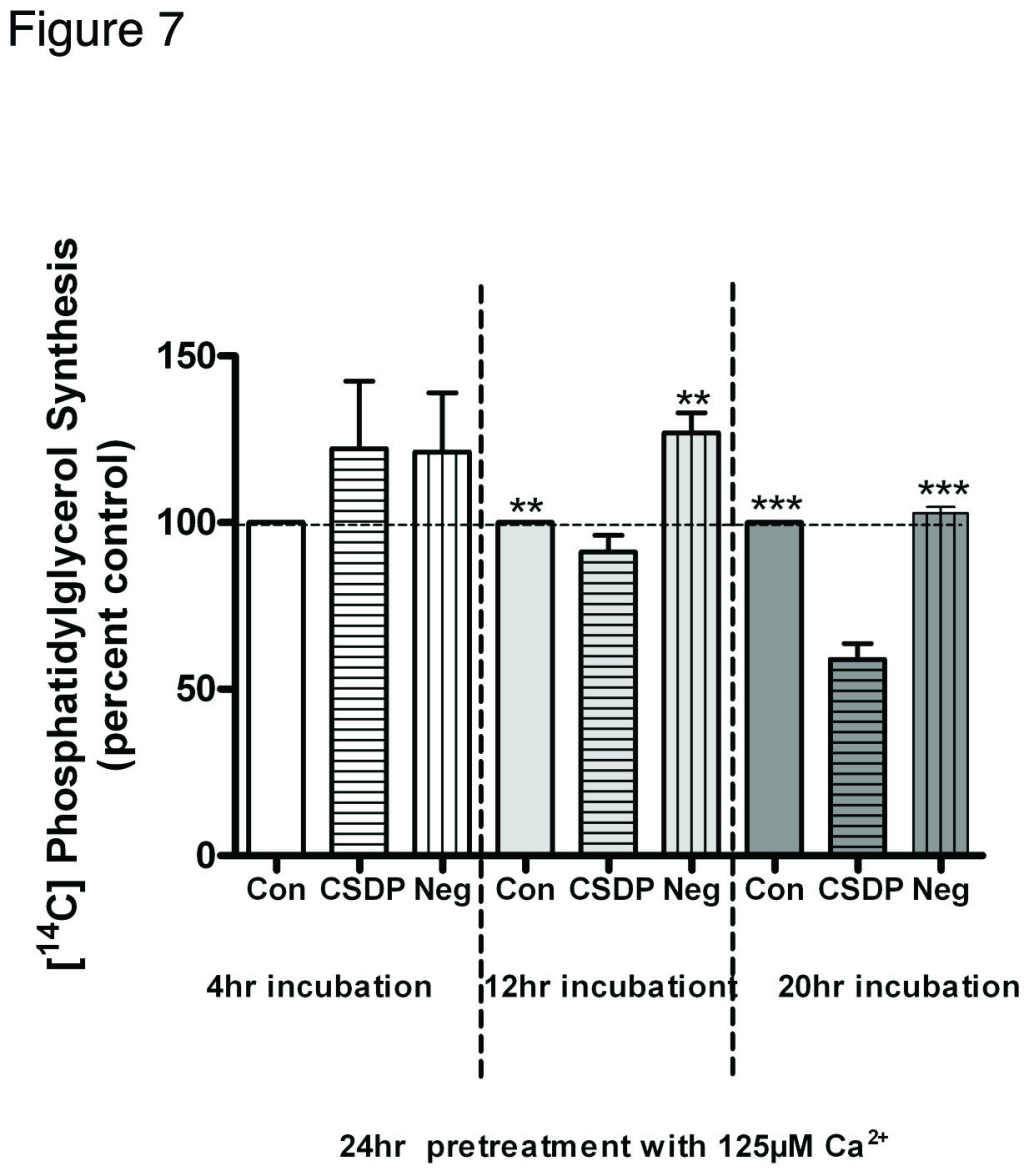
The caveolin-1 scaffolding domain peptide decreases phosphatidylglycerol levels in calcium-pretreated keratinocytes. Keratinocytes were pretreated for 24 hours with SFKM containing 125 µM calcium prior to treatment for the indicated times with vehicle (DMSO) or 3µM caveolin-1 scaffolding domain peptide (CSDP) or the negative control (Neg) as indicated. [^14^C]Phosphatidylglycerol levels were then measured as described in the Materials and Methods. Values are expressed as the percent vehicle and represent the means ± SEM of 3 separate experiments performed in duplicate; **p<0.01 versus the control or CSDP; †††p<0.001 versus the control or negative control.

We have shown that simultaneous treatment with the caveolin-1 scaffolding domain peptide upon elevation of medium calcium levels prevented the calcium-induced increase in phosphatidylglycerol levels. To further study the influence of the caveolin-1 scaffolding domain peptide on phosphatidylglycerol, keratinocytes were pretreated with 125 µM calcium for 24 hours, at which time the differentiated morphology was obvious, followed by treatments with vehicle, the caveolin-1 scaffolding domain peptide, or the negative control for various times and subsequent measurement of phosphatidylglycerol levels. As shown in Figure 7, there was no significant difference in phosphatidylglycerol levels among the three groups after 4 hours’ treatment with the peptides. After 12 hours’ treatment, there was also no difference between control and the caveolin-1 scaffolding domain peptide, although phosphatidylglycerol levels were significantly higher in the negative control group than in either the control or caveolin-1 scaffolding domain peptide group. However, after a 20-hour treatment, phosphatidylglycerol levels were significantly inhibited by the caveolin-1 scaffolding domain peptide, suggesting that even in cells in which differentiation has already been induced by an elevated calcium concentration, the caveolin-1 scaffolding domain peptide can with time disrupt the functional interaction between PLD2 and AQP3 in caveolin-rich membrane microdomains. 

## Discussion

In this study we determined that the caveolin-1 scaffolding domain peptide could prevent the inhibition of proliferation and stimulation of differentiation induced by a moderate calcium concentration. Based on the calcium gradient in different layers of the epidermis [54,55], calcium is regarded as an important factor regulating keratinocyte biology [56]. *In vitro*, raising the extracellular calcium concentration is one of the most potent means of stimulating epidermal differentiation. Keratinocyte differentiation is tightly linked to a rise in intracellular free calcium concentration [57], and elevating the extracellular calcium concentration increases cytosolic calcium levels via binding to the calcium-sensing receptor and activating phosphoinositide hydrolysis [57-59], leading to a series of changes in keratinocyte proliferation and differentiation marker expression. Therefore, following our demonstration of the ability of the caveolin-1 scaffolding domain peptide to inhibit the elevated calcium concentration-induced inhibition of keratinocyte proliferation and promotion of keratinocyte differentiation, it was necessary to determine whether the caveolin-1 scaffolding domain peptide inhibits the initiation of the calcium signaling pathway. Our results showing that pretreatment with 3 µM caveolin-1 scaffolding domain peptide (versus vehicle) for 24 hours did not significantly affect calcium-induced IP_3_ production suggest that activation of the calcium-sensing receptor and stimulation of phosphoinositide hydrolysis was not prevented by the caveolin-1 scaffolding domain peptide. However, our data do not necessarily eliminate the possibility that signaling events downstream of the calcium-sensing receptor and phospholipase C activation may be inhibited by the caveolin-1 scaffolding domain peptide.

PLD2 and AQP3 co-localize in caveolin-rich membrane microdomains [60], and a previous study in our laboratory has suggested the possibility that AQP3 and PLD2 work together to regulate keratinocyte proliferation and differentiation [61]. In this study we found that calcium-increased phosphatidylglycerol levels, but not total PLD nor AQP3 activity, were completely inhibited by the caveolin-1 scaffolding domain peptide, consistent with previous reports that PLD2 activity can be regulated by caveolin-1 [46,47]. This result suggests that PLD2 activity is important in keratinocyte differentiation in response to calcium. 

An important question is the mechanism by which the caveolin-1 scaffolding domain peptide acts to regulate the differentiation process. The caveolin-1 scaffolding domain is involved in membrane attachment and oligomerization of caveolin-1 [62,63]. Although AQP3 and PLD2 interact via a protein-protein interaction, the localization of both proteins in caveolin-1-rich membrane microdomains is mediated by lipids [60]. Thus, it seems reasonable to assume that caveolin-1 facilitates the AQP3/PLD2 signaling pathway by enhancing their colocalization in membrane microdomains, presumably by organizing the lipid domains and allowing AQP3/PLD2 complex formation. High levels of the caveolin-1 scaffolding domain peptide entering the cell membrane can presumably compete with the endogenous full-length caveolin-1 in a time-dependent manner to inhibit membrane attachment and/or oligomerization, thus disrupting the normal function of this protein in membrane microdomains. As a result, even though AQP3 transports similar amounts of glycerol, these glycerol molecules likely have reduced access to PLD2, resulting in an inability of elevated calcium to increase phosphatidylglycerol levels. 

Caveolin-1 has been shown to interact with connexin 43 and regulate gap junctional intercellular communication in keratinocytes [64]. In addition, caveolin-1 has been demonstrated to bind to desmoglein-2 (and desmoglein-1). These proteins partially colocalize in lipid rafts in keratinocytes, and a caveolin-1 scaffolding domain peptide that also binds to desmoglein-2 disrupts the integrity of epidermal sheets *in vitro* [65]. Caveolin-1 has also been found to regulate epidermal tumor formation. In a caveolin-1 knockout mouse model, treatment with a carcinogen, 7,12-dimethylbenzanthracene (DMBA), induced severe epidermal hyperplasia of both the basal and suprabasal cell layers in the null mice in comparison with the wild-type animals [66]. The knockouts also exhibited increased formation of tumors. A second article reported enhanced tumor promotion in caveolin-1 knockout mice as well [67]. Similarly, mammary tumor samples derived from caveolin-1 knockouts crossed with mouse mammary tumor virus-polyoma middle T antigen [PyMT/Cav-1(-/-)] mice show ERK-1/2 hyperactivation, cyclin D1 up-regulation, and Rb hyperphosphorylation, consistent with dysregulated cell proliferation. Interestingly, in this study the addition of the caveolin-1 scaffolding domain peptide is sufficient to inhibit invasion [68], suggesting that under some conditions the caveolin-1 scaffolding domain peptide can mimic caveolin-1 action, presumably by interacting with and regulating the activity of certain signaling enzymes. 

In contrast to the results in mammary epithelium, however, in our study the caveolin-1 scaffolding domain peptide reversed the keratinocyte differentiation induced by moderately elevated calcium, correlating with the epidermal phenotype of the caveolin-1 knockout mouse and the loss of differentiation capacity of suprabasal cells in carcinogen-treated animals [66]. Our results thus suggest a possible dominant negative function of the caveolin-1 scaffolding domain peptide in keratinocytes; such an action might be expected based on the fact that caveolin-1 organizes membrane microdomains and signaling platforms by forming oligomers (reviewed in 36). Thus, if membrane microdomain organization is required for modulation of a particular signaling event, rather than direct interaction of caveolin-1 with certain signaling enzymes, the caveolin-1 scaffolding domain peptide should not substitute for caveolin-1 and could, in fact, act to inhibit the ability of caveolin-1 to stimulate terminal differentiation and act as a “brake” on epidermal proliferation [69]. In this scenario, the requirement for dissociation of caveolin-1 multimers to allow interaction of the caveolin scaffolding domain peptide with caveolin-1 oligomers could explain the relatively long treatment time (20 h) with the caveolin-1 scaffolding domain peptide that was required in order to observe a decrease in phosphatidylglycerol levels in cells pre-differentiated with an elevated extracellular calcium level. This interpretation is also consistent with the finding that caveolin-1 levels are decreased in lesional epidermis of psoriasis [70], a skin disease characterized by hyperproliferation of keratinocytes.

Caveolin-1 binds to and inhibits the activity of multiple signaling molecules, including the epidermal growth factor receptor (EGFR). Traditionally, EGFR signaling has been thought primarily to promote proliferation of keratinocytes; indeed, a mouse model with targeted disruption of EGFR exhibits hypoproliferative epidermis [71]. However, the inhibited differentiation of transgenic mice overexpressing a dominant-negative EGFR in the epidermis [72] suggests that EGFR-mediated signaling also plays a role in epidermal differentiation. Thus, in this study it is possible that the caveolin-1 scaffolding domain peptide inhibited the calcium-induced decrease in proliferation and increase in differentiation through direct inhibition of the EGFR signaling pathway. However, the caveolin-1 scaffolding domain peptide has a lower potency for inhibition of EGFR-mediated signaling than wild-type caveolin-1 [63]. In addition, the similarity between the epidermal phenotype of the caveolin-1 knockout and the caveolin-1 scaffolding domain peptide-treated keratinocytes suggests that the peptide is more likely functioning in a dominant-negative manner, possibly by inhibiting organization of membrane microdomains important in signal transduction processes. 

Our study suggests the possible involvement of PLD2 in elevated calcium concentration-induced keratinocyte differentiation via its formation of phosphatidylglycerol. Our results also suggest multiple ways by which keratinocytes could fine-tune differentiation: by modulating PLD2 or AQP3 expression, their activities or their functional association. However, the mechanism by which keratinocyte differentiation is regulated by PLD2 and phosphatidylglycerol is still unknown. It is possible that PLD2 can activate several protein kinase C isoforms through various lipid messengers (e.g., phosphatidic acid, diacylglycerol and/or lysophosphatidic acid), including protein kinase Cζ [73,74]. As to the likely role of phosphatidylglycerol as a second messenger, there is evidence in the literature to suggest possible effectors. For instance, in fibroblasts phosphatidylglycerol has been identified as a protein kinase C-βII activating factor required for progression to mitosis [75]. Indeed, our initial data suggest that phosphatidylglycerol-activated protein kinase C-βII can alter keratin 10 intermediate filaments (L Bailey and WB Bollag, unpublished data).

In summary, the caveolin-1 scaffolding domain peptide blocked calcium-induced differentiation, as measured by its inhibition of the calcium-elicited decrease in DNA synthesis and increase in transglutaminase activity. The caveolin-1 scaffolding domain peptide blocked the calcium-induced increase in phosphatidylglycerol levels (even in cells pre-differentiated with calcium), with minimal effects on AQP3 or total PLD activities. Thus, our data indicate that the caveolin-1 scaffolding domain peptide inhibits differentiation induced by a moderate increase in extracellular calcium concentration. Further, the ability of this peptide to decrease phosphatidylglycerol levels mediated by the PLD2/AQP3 signaling module, together with the effect of this module to promote keratinocyte differentiation [61], suggest the possibility that the reduction in phosphatidylglycerol levels underlie, at least in part, the inhibition of calcium-induced inhibition of proliferation and promotion of differentiation.
